# 

**Published:** 1864-05

**Authors:** 


					states
IBMOiL m SlfRGim JflMNIL
PRICE, $10 A YEAR Jtf. ADVANCE.

				

